# Cancer co‐medication quality in older patients with polypharmacy: A large randomized trial testing a medication review versus usual care

**DOI:** 10.1111/joim.70161

**Published:** 2026-09-23

**Authors:** Miriam Degen, Annette Kopp‐Schneider, Tomislav Vlaski, Sigrid Carlsson, Peter Hallermann, Ludmilla Jewlanowa, Jana Barinoff, Valentin Goede, Christian Lüders, Thomas Kiefer‐Trendelenburg, Diana Lüftner, Jasmin Doll, Jürgen Wagner, Birgit Leibbrand, Hermann Brenner, Ben Schöttker

**Affiliations:** ^1^ Division of Clinical Epidemiology of Early Cancer Detection German Cancer Research Center (DKFZ) Heidelberg Germany; ^2^ Faculty of Medicine University of Heidelberg Heidelberg Germany; ^3^ Division of Biostatistics German Cancer Research Center (DKFZ) Heidelberg Germany; ^4^ Department of Translational Medicine Division of Urological Cancers Lund University Lund Sweden; ^5^ Clinic Niederrhein Bad Neuenahr‐Ahrweiler Germany; ^6^ Rehabilitation Centre Ückeritz/Clinic Ostseeblick Ückeritz Germany; ^7^ Department of Oncogeriatrics Geriatric Medicine Center St. Marien Hospital Cologne Germany; ^8^ Klinik am See Rehabilitation Center for Internal Medicine Rüdersdorf bei Berlin Germany; ^9^ Brandenburg Medical School Theodor Fontane (MHB) Brandenburg Germany; ^10^ Immanuel Clinic Märkische Schweiz Buckow Germany; ^11^ Immanuel Clinic Rüdersdorf Medical University of Brandenburg Theodor Fontane (MHB) Rüdersdorf bei Berlin Germany; ^12^ Clinic MEDICLIN Staufenburg Durbach Germany; ^13^ Salzetalklinik Center for Oncological and Orthopedic Rehabilitation Bad Salzuflen Germany; ^14^ Cancer Prevention Graduate School German Cancer Research Center (DKFZ) Heidelberg Germany

**Keywords:** cancer, geriatrics, internal medicine, medication review, quality of healthcare

## Abstract

**Background:**

Evidence from randomized controlled trials (RCTs) testing medication reviews in geriatric oncology is sparse.

**Methods:**

We conducted a single‐blinded RCT in ten rehabilitation clinics across Germany. Patients with common cancers, aged ≥ 65 years, and polypharmacy (regularly ≥ five drugs) were randomly allocated in equal proportions to usual care or a comprehensive pharmacist‐led medication review conducted with the validated Fit fOR The Aged (FORTA) list. The FORTA score is the sum of drugs classified as potentially inappropriate medication (PIM) (each two points), drug underuse (each one point), and drug overuse (each one point). Changes in the FORTA score and its subcomponents from rehabilitation admission to discharge were tested using the chi‐square test or the Wilcoxon's rank‐sum test.

**Findings:**

At admission, 66.1%, 94.6%, and 58.9% of 443 trial participants included in the intention‐to‐treat analysis were exposed to PIM, medication underuse, or overuse, respectively, and the median FORTA score was six. At discharge, participants of the intervention group (*n* = 234) had a statistically significant greater reduction in the mean FORTA score than the control group (*n* = 209): mean difference [95% confidence interval]: −3.7 [−4.2; −3.3] score points. Furthermore, the proportions of subjects with PIM, underuse, and overuse were statistically significantly reduced by the intervention by 41.3, 35.5, and 30.7 percentage points (all *p *< 0.001).

**Interpretation:**

The pharmacist‐led intervention with the FORTA list substantially improved the medication quality of older cancer patients with polypharmacy in clinical practice. A reduction of four FORTA score points (rounded) translates, for example, to the withdrawal of two PIMs or four drugs labeled overuse.

## Graphical abstract

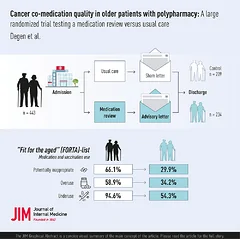

**Abbreviations**
AM: appropriate medication
CI: confidence interval
DACHS: Darmkrebs: Chancen der Verhütung durch Screening
DUFSS‐5: Duke‐UNC Functional Social Support Questionnaire, 5‐item version
EORTC: European Organisation for Research and Treatment of Cancer
FORTA: Fit fOR The Aged
GDS: Geriatric Depression Scale
GP: general practitioner
IQR: interquartile range
NSAIDs: nonsteroidal anti‐inflammatory drugs
OPTIMAL: optimization of polypharmacy in geriatric oncology
PIM: potentially inappropriate medication
PPI: proton pump inhibitors
QLQ: quality of life questionnaire
RAPA: Rapid Assessment of Physical Activity
RCT: randomized controlled trial
VIF: variance inflation index

## Background

How to ensure optimal cancer and noncancer pharmacotherapy for older adults is a critical question facing clinicians in our aging and increasingly multimorbid [1] society. Specifically, evidence from the German Darmkrebs: Chancen der Verhütung durch Screening (DACHS) study demonstrated that approximately half of the colorectal cancer patients aged 65 years or older were exposed to polypharmacy (≥ five medications) and even more than nine out of ten experienced medication problems, which were statistically significantly inversely associated with overall and cancer‐specific survival [2, 3]. Hence, there is a pressing need for effective and feasible medication optimization strategies in older cancer patients.

Medication problems comprise underuse and overuse of medications as well as so‐called potentially inappropriate medications (PIM) with an expected poor benefit‐to‐harm ratio in geriatric patients due to established associations with hospitalizations and premature death [4, 5, 6, 7]. Several drug lists exist to identify medication problems among older adults, and a comprehensive, validated tool for the German drug market is the Fit fOR The Aged (FORTA) list [5]. Based on regularly updated evidence and expert opinions, the FORTA classes A (indispensable) to D (avoid) inform about appropriate medications (AM) and PIM for 30 noncancer and cancer indications. The FORTA list is also suitable for assessing drug underuse and overuse, and all three medication problem sub‐categories can be combined into the total FORTA score as a measure of overall medication quality [5].

FORTA‐based medication reviews hold potential promise as a feasible, effective strategy to optimize cancer and co‐medication quality (and mitigate associated risks) for older cancer patients in clinical practice. This hypothesis is based not only on evidence from the aforementioned DACHS study [2, 3] but also on a randomized controlled trial (RCT) demonstrating the applicability and effectiveness of this freely accessible tool in general geriatric hospital wards [8]. However, high‐level evidence is still lacking for older cancer patients with polypharmacy, who are particularly vulnerable.

The three‐week inpatient rehabilitation, which is part of usual care shortly after completion of the primary oncologic therapy in Germany [9], represents an ideal context to conduct a comprehensive medication review because usually several changes have been made to the cancer and co‐medication in hospitals or specialist practices during cancer treatment, and the general practitioner (GP) may not (yet) be aware of them.

Therefore, this investigation aims to evaluate the superiority of an advisory letter, with the results of a FORTA‐based, pharmacist‐led medication review sent to the treating rehabilitation physician, compared with usual care, on the medication‐quality‐related secondary outcomes of the “Optimization of polypharmacy in geriatric oncology” (OPTIMAL) trial in older cancer patients with polypharmacy. As the follow‐up of the OPTIMAL study is still ongoing, the primary endpoint, “Quality of life at 8‐month follow‐up,” will be published separately later.

## Materials and methods

### Trial registration and reporting

This investigation was reported according to the CONSORT guideline [10] and follows the published protocol of the OPTIMAL trial [11], which has additionally been registered in the German Clinical Trials Register (no. DRKS00031024).

### Trial design, inclusion, and exclusion criteria

The parallel‐group, randomized, controlled OPTIMAL trial assesses the efficacy of a pharmacist‐led medication review in older cancer patients exposed to polypharmacy during inpatient rehabilitation compared to usual care (no pharmacist‐led medication review). The study is single‐blinded, as only patients were fully blinded to the allocation. The study was approved by the Ethics Committee of the Medical Faculty, Heidelberg University (08/02/2023), and adhered to the Declaration of Helsinki.

Participants aged ≥ 65 years, exposed to polypharmacy (≥ five regularly used drugs), with a cancer diagnosis or recurrence (22 common types) within five years before the beginning of their rehabilitation, and an expected inpatient stay of three weeks in one out of ten collaborating German rehabilitation clinics were eligible for inclusion. Exclusion criteria encompassed no written informed consent, no medication‐related problem at baseline (FORTA score = 0), insufficient mental and physical capabilities to fill out questionnaires, insufficient German language knowledge, and missing information needed to conduct the medication review. Specifically for this investigation, we further excluded trial participants without information from a checklist about medication changes made by the rehabilitation physician. This checklist was introduced approximately five months after the start of recruitment (on 26/10/2023), when we noticed that the routine discharge documentation was not detailed enough.

### Recruitment and randomization

A randomization list with allocation in equal distributions to the medication review and usual care group was prepared by the principal investigator in the coordinating center (Division of Clinical Epidemiology of Early Cancer Detection, German Cancer Research Center) before the start of the study with randomizer.at, using permuted blocks of random sizes stratified by rehabilitation clinic. Study materials were labeled with the randomization IDs and shipped to the rehabilitation clinics. Between June 2023 and September 2025, rehabilitation physicians recruited participants shortly after their admission to the rehabilitation clinic and randomized them by picking the pre‐labeled study materials. Except for the study pharmacists in the coordinating center, no study physician, study personnel, or participant had access to the allocation sequence.

### First study visit and collection of baseline information

During the first medical appointment with their treating rehabilitation physician, participants provided written informed consent and received a baseline questionnaire about socio‐demographic information, lifestyle factors, medical history (including over‐the‐counter medication), current symptoms (e.g., pain, insomnia, nausea), medication use, and validated questionnaires for social support (Duke‐UNC Functional Social Support Questionnaire (DUFSS‐5) [12]), physical activity (Rapid Assessment of Physical Activity (RAPA) tool [13]), frailty (FRAIL scale [14]), depression (Geriatric Depression Scale [15]), and quality of life (QLQ, European Organisation for Research and Treatment of Cancer QLQ‐C30 [16]).

Additionally, the study physicians provided relevant cancer‐related information (International Classification of Diseases, 10th revision code, stage, and primary treatment), medication use, and medical diagnoses through a checklist and/or copies of electronic health records. All study documents were sent to the coordinating center.

### FORTA‐based medication review

Upon receipt of the study documents, a study pharmacist at the coordinating center created a complete list of medical diagnoses and medication use, compiling information from physicians and patients. The pharmacist subsequently conducted the medication review for the intervention group using the fourth version of the FORTA list [5], the most recent at the time of study initiation (last updated in 2021). In this version of the FORTA list, vaccine underuse was added as an additional domain.

The medication review assessed three categories of medication problems and assigned points:

1. PIM (two points per drug): Medications classified as C (questionable) or D (avoid) for a specific indication of the 30 FORTA indications were recorded as PIM.
2. Overuse (one point per drug): For all remaining medications, the pharmacist reviewed whether the medication was indicated to treat an additional indication not included in the 30 FORTA indications. Appropriate use of medications not listed on the FORTA list was determined according to clinical guidelines. Each medication without an appropriate indication was recorded as overuse.
3. Underuse (one point per untreated indication or vaccination underuse): Each untreated FORTA indication was recorded as medication underuse. In addition, vaccination underuse for herpes zoster, pneumococcus, influenza (in the last 12 months), and SARS‐CoV2 (vaccination or infection in the last 12 months) was assessed by self‐reported information.

The total FORTA score sums up the points for PIM, underuse, and overuse. We calculated it with and without points for vaccination underuse. For participants allocated to the medication review, the pharmacist created an advisory letter with recommendations to switch PIM to appropriate alternatives for the respective indication classified as A (indispensable) or B (beneficial) by the FORTA list. Moreover, the letter provided recommendations to initiate appropriate drugs (with FORTA classification A or B) for untreated indications, to administer missing vaccinations, and to discontinue medications classified as “overuse”.

All proposed new drugs were checked for potential drug–drug interactions and contraindications, and recommendations were adapted as needed. Additional advice was included where appropriate (e.g., dosage adjustments, monitoring of specific parameters).

The whole process of the medication review and creation of the recommendation letters was automated with a statistical analysis software (SAS) code. The pharmacist only checked the automatically generated recommendation letters for plausibility, contraindications, and potential drug–drug interactions. Initial mistakes made by the SAS code were used to improve it over the course of the study, and it was not changed anymore after about 100 medication reviews.

For participants allocated to the usual care group, the pharmacist created a sham advisory letter stating that no medication recommendations were suggested. To keep the study physicians partially blinded, no information about the group allocation was included. However, trial physicians knew the text of the sham letters from the study protocol and could guess the group allocation correctly in most cases because there were very few study participants in the intervention group for whom no medication recommendations were suggested.

The final version of the SAS code was used to automatically calculate the FORTA score and its sub‐scores at rehabilitation admission and discharge for both the intervention and control groups.

### Second study visit and documentation of medication changes in the checklist

Study physicians were free to decide which recommendations they would accept and/or discuss with the patient during the second study visit, which took place in the last week of rehabilitation. Accepted changes could be implemented directly by the rehabilitation physician or communicated to the patient's GP via the discharge letter. Each accepted and rejected recommendation in the advisory letter had to be documented on a checklist, along with updated information on medical diagnoses and medication use at the time of rehabilitation discharge.

Rehabilitation physicians were allowed to make medication changes as part of usual care for all trial participants. This fact was used to blind the study participants. Physicians informed the participants about possible medication changes, explaining that any of these could result either from their own medical judgment as part of routine care or from allocation to the intervention group of the OPTIMAL study, but the true reason was not revealed. Not discussing medication changes could imply either optimal medication quality or assignment to the usual care group. The exact wording for the discussion with patients was specified in the advisory letter and sham letter to avoid accidental unblinding of the participants. The physicians were instructed to conduct a second study visit with all study participants, even if they received the sham letter, and to document that the meeting took place. This should ensure that all study participants were treated equally by study physicians and remained blinded to the intervention.

### After rehabilitation clinic discharge

The pharmacist at the coordinating center used the medication and diagnostic information from the checklist to calculate the FORTA score at rehabilitation discharge. If the GP was asked to implement the medication changes or administer vaccinations in the discharge letter, these were treated as implemented recommendations for the FORTA score calculation at discharge.

We also calculated the FORTA score in the usual care group to assess potential learning effects of physicians during the trial, as specified in the trial protocol [11]. However, no learning effects were observed.

### Outcomes analyzed in this investigation

As the follow‐up of the OPTIMAL study is still ongoing, the primary endpoint, “Quality of life at 8‐month follow‐up,” will be published later, along with a follow‐up on the maintenance of medication changes after discharge by the GPs. This investigation addresses the predefined secondary endpoints of medication quality until rehabilitation discharge. The main outcome of interest is the change in total FORTA score points from rehabilitation admission to discharge. Further secondary endpoints include changes in FORTA sub‐scores and proportions of PIM use, underuse, overuse, and any of the aforementioned medication problems.

The frequency and acceptance rates of recommendations to reduce overuse and PIM use per medication class and to reduce underuse per indication in the intervention group were calculated from the returned checklists. Specifically for overuse and underuse, patients sometimes forgot to mention a previous disease or a medication at baseline, which turned out while discussing medication changes during the second study visit. Such resolved medication problems were not directly attributable to the intervention. Therefore, we only counted resolved medication problems attributable to the intervention (accepted switch, drug initiation, or discontinuation) as accepted.

### Statistical analyses

As the FORTA (sub‐)scores at discharge appeared to be not normally distributed and the changes in FORTA (sub‐)scores were approximately normally distributed (see histograms in Figs. S1 and S2, respectively), the Wilcoxon rank‐sum test was used as the primary test statistic for FORTA (sub‐)scores at discharge and the *t* test for changes in FORTA (sub‐)scores. The 95% confidence intervals (95% CI) were estimated using quantile regression with resampling for non‐normally distributed outcomes and the Welch–Satterthwaite method for normally distributed outcomes. Chi‐square tests were used to compare the proportions of patients exposed to PIM, medication overuse, underuse, and any of these three medication problems. If the counts for an exposure were < 5, Fisher's exact test was used instead of the chi‐square test. The 95% CIs were estimated using Wald methods. We applied a significance level of *α* = 0.05 for all statistical tests. As the outcomes in this manuscript are secondary endpoints, all other results should be regarded as exploratory and not confirmatory.

In a sensitivity analysis, we estimated β‐coefficients with 95% Wald CIs for the treatment group allocation with respect to the changes in FORTA scores and their sub‐scores from admission to discharge using a multivariable adjusted linear regression model. Additionally, to account for the stochastic dependence of results among study physicians, cluster‐robust standard errors were estimated with study physicians as the clustering variable using the cluster statement in the SAS procedure “PROC SURVEYREG”. Linearity, independence, homoscedasticity, and multicollinearity were assessed prior to the analysis, with no violation detected. The models were adjusted for 38 covariates, including the baseline FORTA score or the respective sub‐score, age, sex, education, partnership, social support, body mass index, alcohol consumption, physical activity, smoking status, cancer site, cancer stage, previous cancer therapy, frailty, global quality of life, depressive symptoms, and 20 common indications as single covariates (as listed in Table 1). Multicollinearity and model stability were assessed using the variance inflation index (VIF), and all variables had VIF ≤ 3.8.

**Table 1:** Baseline characteristics of the intention‐to‐treat population (*N* = 443).

	Control (*N* = 209)		Intervention (*N* = 234)	
Characteristics	*N* (%)^a^	Mean (SD)^a^	*N* (%)^a^	Mean (SD)^a^
***Socio‐demographic factors***				
Sex				
Female	147 (70.3)		155 (66.2)	
Male	62 (29.7)		79 (33.8)	
Age		73.5 (6.0)		73.2 (5.7)
Years of schooling				
≤9	83 (39.7)		83 (35.5)	
10–11	87 (41.6)		94 (40.2)	
≥12	39 (18.7)		57 (24.4)	
Partnership				
Yes	129 (61.7)		157 (67.1)	
No	80 (38.3)		77 (32.9)	
Social support^b^		16.0 (5.0)		16.0 (5.3)
***Lifestyle factors***				
BMI (kg/m^2^)		28.2 (5.8)		27.7 (5.2)
Alcohol consumption^c^ (g/day)				
Abstainer	79 (37.8)		83 (35.5)	
WHO class I	126 (60.3)		143 (61.1)	
WHO class II–III	4 (1.9)		8 (3.4)	
Physical activity^d^				
Sedentary or underactive	19 (9.1)		25 (10.7)	
Regular underactive, light activities	54 (25.8)		51 (21.8)	
Regular underactive	61 (29.2)		88 (37.6)	
Regular active	75 (35.9)		71 (30.3)	
Smoking status				
Never smoker	117 (56.0)		124 (53.0)	
Former				
<20 years	27 (12.9)		37 (15.8)	
≥20 years	52 (24.9)		55 (23.5)	
Current	13 (6.2)		18 (7.7)	
***Cancer‐related factors***				
Cancer site^e^				
Breast	111 (53.1)		117 (50.0)	
Prostate	31 (14.8)		40 (17.1)	
Other	67 (32.1)		77 (32.9)	
Stage				
I	91 (43.5)		108 (46.2)	
II	55 (26.3)		59 (25.2)	
III	52 (24.9)		40 (17.1)	
IV	13 (6.2)		30 (12.8)	
Previous cancer therapy				
Surgery	183 (87.6)		201 (85.9)	
Chemotherapy	69 (33.0)		75 (32.1)	
Radiotherapy	114 (54.5)		128 (54.7)	
***Comorbidities***				
Diabetes	67 (32.1)		70 (29.9)	
Arterial hypertension	188 (90.0)		204 (87.2)	
Stroke	25 (12.0)		30 (12.8)	
Myocardial infarction	34 (16.3)		32 (13.7)	
Heart failure	31 (14.8)		42 (17.9)	
Acute coronary syndromes	28 (13.4)		35 (15.0)	
Atrial fibrillation	59 (28.2)		51 (21.8)	
Asthma, COPD	43 (20.6)		43 (18.4)	
Osteoporosis	53 (25.4)		50 (21.4)	
Chronic pain^f^	165 (78.9)		172 (73.5)	
History of depression	44 (21.1)		26 (11.1)	
Insomnia	47 (22.5)		63 (26.9)	
Gastrointestinal illnesses^g^	29 (13.9)		33 (14.1)	
Incontinence	54 (25.8)		75 (32.1)	
Anaemia^h^	29 (13.9)		39 (16.7)	
Constipation	15 (7.2)		12 (5.1)	
Hypothyroidism	80 (38.3)		97 (41.5)	
Chronic kidney disease	39 (18.7)		47 (20.1)	
Dyslipidemia	37 (17.7)		36 (15.4)	
Peripheral vascular disease	12 (5.7)		16 (6.8)	
***Frailty*** ^i^				
No	66 (31.6)		82 (35.0)	
Pre‐frail	86 (41.1)		117 (50.0)	
Frail	57 (27.3)		35 (15.0)	
***Quality of life (QoL)***				
Global QoL score^j^		52.4 (18.2)		53.8 (17.0)
***Symptoms***				
Depressive symptoms^k^	79 (37.8)		78 (33.3)	
***Exposure*** ^l^				
Total FORTA score				
Incl. vaccine underuse	209 (100.0)	6.6 (2.9)	234 (100.0)	6.2 (3.0)
Without vaccine underuse	209 (100.0)	4.6 (2.6)	224 (95.7)	4.3 (2.8)
FORTA score sub‐categories				
PIM	150 (71.8)	2.6 (2.6)	143 (61.1)	2.3 (2.6)
Overuse	123 (58.9)	0.9 (1.0)	138 (59.0)	0.9 (1.0)
Underuse				
Incl. vaccine underuse	195 (93.3)	3.1 (1.7)	224 (95.7)	3.0 (1.6)
Without vaccine underuse	152 (72.7)	1.1 (0.9)	163 (69.7)	1.1 (1.0)

*Note*: Proportions in certain categories do not sum up to 100.0% due to rounding issues.

Abbreviations: BMI, body mass index; COPD, chronic obstructive pulmonary disease; FORTA, Fit fOR The Aged List Version 4; QoL, quality of life; SD, standard deviation; WHO, World Health Organization.

^a^ Calculated from first imputed dataset.

^b^ According to DUFSS‐5 [12].

^c^ In 12 months before cancer diagnosis.

^d^ According to Rapid Assessment of Physical Activity (RAPA) tool [13].

^e^ Primary cancer according to inclusion criteria.

^f^ Combination of self‐reported pain in the last week and pain‐related diagnoses (including migraine, other headache, chronic pain syndromes, arthritis, osteoarthritis, gout, and dorsopathies).

^g^ Including regular use of nonsteroidal anti‐inflammatory drugs.

^h^ Due to iron or vitamin B12 deficiency.

^i^ According to FRAIL scale [14].

^j^ According to the EORTC QLQ‐C30 global health status/quality of life scale [16].

^k^ According to the GDS [15].

^l^ Recommendations according to the “The FORTA (Fit fOR The Aged) List Version 4”.

We used SAS statistical software (version 9.4, SAS Institute, Inc.) for the statistical analyses. We used multiple imputation with the Markov Chain Monte Carlo method to impute missing covariate and endpoint values stratified by trial group, creating 20 imputed datasets, which were subsequently pooled using Rubin's rule with the SAS procedure “PROC MIANALYZE”. The FORTA score at discharge was missing for *n* = 25 (5.6%) of the study participants. The cancer stage was unknown for 12.4% of study participants, and no other covariate had more than 2.5% of missing values (Table S1). All variables from the multivariable linear regression model were used for the imputation model, and it was extended for the rehabilitation clinic and the FORTA sub‐scores at baseline. Further details about the multiple imputation procedure are outlined in Text S1.

This investigation was analyzed using an intention‐to‐treat approach, with every patient correctly included in the trial analyzed according to the group to which they were randomized. Additional per‐protocol analyses were conducted, excluding all participants who had no documented second study visit and all participants assigned to the intervention group who did not return a discharge checklist. Subgroup analyses were conducted by rehabilitation clinic, sex, and age groups (65–74 and ≥ 75 years). Clinics that recruited fewer than 50 patients were combined. All statistical analyses were prespecified in the statistical analysis plan. No interim analyses were conducted.

### Patient involvement

The relevance and appropriateness of the study were discussed with and confirmed by a representative of a patient advocacy group (Deutsche ILCO e.V.) prior to study initiation.

## Results

### Characteristics of the study population

Figure S3 shows the study flow‐chart. Out of *n* = 561 individuals randomized between 05/06/2023 and 08/09/2025, *n* = 542 were correctly included in the trial because they met the inclusion criteria and did not withdraw consent during the trial. These study participants were recruited by 23 physicians in ten rehabilitation clinics. *n* = 443 (*n*_intervention_, *n* = 234; *n*_control_ = 209) of these participants were eligible for inclusion in the intention‐to‐treat analysis of this investigation because they were recruited after 26/10/2023, when the discharge checklist was implemented. Thereof, we excluded *n* = 17 with a missing discharge checklist and *n* = 8 with completely missing documentation of the second study visit (consideration of the intervention with the advisory/sham letter by study physician is unlikely) for the per‐protocol analysis, leaving *n* = 418 participants (*n*_intervention_, *n* = 212; *n*_control_, 206).

In the total study population, the mean age was 73.3 years (standard deviation: 5.9), and 68.2% of the population was female. Table 1 presents the baseline characteristics of the study population separately for the control and intervention groups. Except for a history of depression and frailty, which were more common in the control group, baseline characteristics were well balanced between the study arms.

### Medication quality at admission to the rehabilitation clinics

In the total study population at admission, the median (interquartile range (IQR)) FORTA score was 6 (4; 8) with and 4 (3; 6) without counting vaccine underuse (Table S2). The median FORTA sub‐scores indicate that study participants had, on average, one PIM, used one drug without an indication (overuse), one FORTA‐indicated drug missing (medication underuse), and two recommended vaccinations missing (vaccination underuse). The proportions of patients exposed to PIM, medication overuse, and medication underuse were 66.1%, 58.9%, and 71.1%, respectively. There were no relevant differences between the trial arms (Table 1).

### Change in medication quality from admission to discharge

Box and scatter plots to display the distribution of the total FORTA score and its sub‐scores at admission and discharge (Fig. S4), as well as for their changes from admission to discharge (Fig. S5), are presented in the Supporting Information. The median (IQR) total FORTA score in the control group (6 (4; 9)) was statistically significantly higher at discharge compared to the median in the intervention group (2 (0; 4)), corresponding to a median difference (95% CI) of −4 (−4.8; −3.2) score points (Table 2). Consequently, we observed a similar, statistically significant mean difference (95% CI) in change in total FORTA score of −3.7 (−4.2; −3.3) score points from admission to discharge in the intervention compared to the control group (*p *< 0.001). Not considering vaccines would lead to a one‐point lower change in the mean (95% CI) difference of the total FORTA score (−2.6 (−3.0; −2.2)), which was statistically significant as well. The FORTA sub‐scores for PIM, overuse, and underuse (including or excluding vaccines) were also statistically significantly reduced (all *p* < 0.001). The sensitivity analyses with a multivariable linear regression model (Table S3) and with cluster‐robust estimators for the standard errors (Table S4) yielded results almost identical to those of the main analysis. The results were consistent in the per‐protocol analysis (Table S5).

**Table 2:** Median difference in FORTA (sub‐)score scales between intervention and control group at discharge and mean difference in changes from admission to discharge in intention‐to‐treat analysis (*N* = 443).

	At discharge				Change from admission to discharge			
FORTA score^a^	Control (*n* = 209) Median (IQR)	Intervention (*n* = 234) Median (IQR)	Median Difference (95% CI)	*p*	Control (*n* = 209) Mean (95% CI)	Intervention (*n* = 234) Mean (95% CI)	Difference Mean (95% CI)	*p*
**Total**								
Incl. vaccine underuse	6 (4; 9)	2 (0; 4)	**−4 (−4.8; −3.2)**	**<0.001**	−0.1 (−0.3; 0.1)	**−3.9 (−4.3; −3.5)**	**−3.7 (−4.2; −3.3)**	**<0.001**
Without vaccine underuse	4 (3; 6)	1 (0; 3)	**−3 (−3.6; −2.4)**	**<0.001**	0.2 (0.1; 0.4)	**−2.3 (−2.7; −2.0)**	**−2.6 (−3.0; −2.2)**	**<0.001**
**Sub‐categories**								
PIM	2 (0; 4)	0 (0; 2)	**−2 (−2.0; −2.0)**	**<0.001**	0.0 (−0.1; 0.1)	**−1.4 (−1.7; −1.1)**	**−1.4 (−1.8; −1.1)**	**<0.001**
Overuse	1 (0; 1)	0 (0; 1)	**−1 (−1.0; −1.0)**	**<0.001**	0.1 (0.1; 0.2)	**−0.5 (−0.6; −0.4)**	**−0.6 (−0.8; −0.5)**	**<0.001**
Underuse								
Incl. vaccine underuse	3 (1; 4)	1 (0; 2)	**−2 (−2.5; −1.5)**	**<0.001**	−0.3 (−0.4; −0.1)	**−2.0 (−2.2; −1.7)**	**−1.7 (−2.0; −1.4)**	**<0.001**
Without vaccine underuse	1 (0; 2)	0 (0; 1)	**−1 (−1.0; −1.0)**	**<0.001**	0.1 (0.0; 0.2)	**−0.4 (−0.6; −0.3)**	**−0.6 (−0.7; −0.4)**	**<0.001**

*Note*: Values in bold are statistically significant (*p* < 0.05).

Abbreviations: CI, confidence interval; FORTA, Fit fOR The Aged List Version 4; IQR, interquartile range; PIM, potentially inappropriate medication.

^a^ Recommendations according to the “The FORTA (Fit fOR The Aged) List Version 4”.

On the individual participant level, the proportion of study participants exposed to any medication problem was statistically significantly (*p *< 0.001) 25.2 percentage points lower at discharge in the intervention group compared to the control group, in which all participants (100.0%) remained exposed (Table 3). The highest absolute exposure difference at discharge was observed for PIM (−41.3 percent points), followed by underuse including vaccines (−35.5 percent points) and overuse (−30.7% percent points), which were all highly statistically significant (*p* < 0.001). In the per‐protocol analysis, the reduction was approximately 2 percentage points greater in each category (Table S6).

**Table 3:** Proportions of study participants exposed to medication problems at discharge in the intention‐to‐treat population (*N* = 443).

Medication problems^a^	Control (*n* = 209) *N* (%)^b^	Intervention (*n* = 234) *N* (%)^b^	Absolute difference^c^ (95% CI)	*p*
**Any (FORTA score > 0)**				
Incl. vaccine underuse	209 (100.0)	175 (74.8)	**−25.2 (−30.9; −19.5)**	**<0.001** ^d^
Without vaccine underuse	209 (100.0)	172 (73.5)	**−26.6 (−32.4; −20.8)**	**<0.001** ^d^
**FORTA sub‐categories**				
PIM	148 (70.8)	70 (29.9)	**−41.3 (−50.1; −32.6)**	**<0.001**
Overuse	134 (64.1)	80 (34.2)	**−30.7 (−39.7; −21.7)**	**<0.001**
Underuse				
Incl. vaccine underuse	187 (89.5)	127 (54.3)	**−35.5 (−43.5; −27.6)**	**<0.001**
Without vaccine underuse	153 (73.2)	105 (44.9)	**−26.7 (−35.8; −17.6)**	**<0.001**

*Note*: Values in bold are statistically significant (*p* < 0.05).

Abbreviations: CI, confidence interval; FORTA, Fit fOR The Aged List Version 4; PIM, potentially inappropriate medication.

^a^ Recommendations according to the “The FORTA (Fit fOR The Aged) List Version 4”.

^b^ Rounded values from first imputed dataset.

^c^ In proportions exposed to the relevant medication problem.

^d^ Tested with Fisher's exact test as all participants of the control group were exposed.

The subgroup analysis by rehabilitation clinics showed that the two clinics that recruited the most study participants also implemented more medication changes from the recommendation letters than the other clinics (Table S7). Nevertheless, a significant reduction in FORTA scores from admission to discharge was also observed in the clinic that ranked third in recruitment and all the other seven clinics combined. The subgroup analyses by sex and age groups yielded similar implementation rates for female and male patients (Table S8) as well as for those aged 65–74 and 75–92 years (Table S9).

### Frequency and acceptance rates of recommendations

Out of 218, 191, and 610 recommendations made to reduce PIM, overuse, and underuse in the intervention group, respectively, the acceptance rates attributable to the intervention were 77.1% for switching PIM, 77.0% for withdrawal of overused drugs, and 75.1% for underuse‐specific recommendations. The latter included 52.4% accepted new drug initiations and 89.9% vaccinations, respectively.

Table 4 shows that most recommendations (≥ 50) regarding changes of PIM were made for analgesics and beta blockers, and that they were accepted in > 75% of cases. Antidepressants, antipsychotics/sedatives, and drugs for obstructive airway diseases were identified as PIM ≥ 20 times, and acceptance rates varied from 65% to 80%. All other PIM drug groups were rather rare (≤ ten recommendations).

**Table 4:** Proportions of accepted recommendations from the advisory letter regarding PIM in the intervention group per medication group (*N* = 212; per‐protocol population).

	Recommendations^a^	Proportion accepted^b^
Medication group	***n***	***n* (%)**
Analgesics^c^	54	43 (79.6)
Beta blockers	50	38 (76.0)
Antidepressants	23	17 (73.9)
Antipsychotics and sedatives^d^	20	16 (80.0)
Drugs for obstructive airway diseases	20	13 (65.0)
Spironolactone	10	8 (80.0)
Antidiabetics	8	8 (100.0)
Antithrombotic agents	6	3 (50.0)
Antihypertensives^e^	4	3 (75.0)
Anti‐incontinence drugs	4	3 (75.0)
Antiarrhythmics	3	3 (100.0)
Antiepileptics	3	3 (100.0)
Corticosteroids, systemic use	3	2 (66.7)
Expectorants, cough suppressants	3	3 (100.0)
Drugs for bone diseases^f^	2	1 (50.0)
Drugs for constipation	2	2 (100.0)
Antiemetics	1	1 (100.0)
Antiparkinson drugs	1	1 (100.0)
Endocrine antineoplastic therapy	1	0 (0.0)
**Total**	**218**	**168 (77.1)**

Abbreviation: PIM, potentially inappropriate medication.

^a^ Recommendations according to the “The FORTA (Fit fOR The Aged) List Version 4”.

^b^ The recommendation to switch a PIM to a recommended appropriate medication was accepted. The medication change was either directly initiated by the study physician or the recommendation was communicated to the patient's general practitioner. All accepted switches were considered attributable to the intervention.

^c^ Including nonsteroidal anti‐inflammatory drugs.

^d^ Including anxiolytics, hypnotics, and antihistamines for systemic use (psycholeptics).

^e^ Excluding beta blockers.

^f^ Including hormone replacement therapy in participants diagnosed with osteoporosis.

Table 5 presents the proportions of accepted recommendations from the advisory letter regarding overuse in the intervention group per medication group. Proton‐pump inhibitors (PPIs) were the most common drug group classified as overuse (*n* = 41 recommendations), followed by lipid‐modifying agents, antithrombotic agents, analgesics, and antipsychotics/sedatives, with 14–28 recommendations each. The acceptance rate to discontinue these drug classes ranged from 71% to 88%. All other drug classes were rarely classified as overuse (< ten recommendations).

**Table 5:** Proportions of accepted recommendations from the advisory letter regarding overuse in the intervention group per medication group (*N* = 212; per‐protocol population).

	Recommendations^a^	Proportion accepted^b^
Medication group	***n***	***n* (%)**
PPIs	41	35 (85.4)
Lipid‐modifying agents	28	22 (78.6)
Antithrombotic agents	24	21 (87.5)
Analgesics^c^	20	16 (80.0)
Antipsychotics and sedatives^d^	14	10 (71.4)
Antiglaucoma drugs	8	6 (75.0)
Drugs for BHP	8	6 (75.0)
Drugs for constipation	7	1 (14.3)
Iron supplements	7	5 (71.4)
Antiparkinson drugs	5	3 (60.0)
Mineral supplements	4	3 (75.0)
Antidepressants	4	3 (75.0)
Drugs for obstructive airway diseases	4	3 (75.0)
Thyroid preparations	3	2 (66.7)
Antidiabetics	3	2 (66.7)
Drugs for bone diseases	3	2 (66.7)
Organic nitrates	3	3 (100.0)
Diuretics	2	1 (50.0)
Antidementia drugs	1	1 (100.0)
Antiepileptics	1	1 (100.0)
Corticosteroids, systemic use	1	1 (100.0)
**Total**	**191**	**147 (77.0)**

Abbreviations: BHP, benign prostatic hypertrophy; PIM, potentially inappropriate medication; PPIs, proton pump inhibitors.

^a^ Recommendations according to the “The FORTA (Fit fOR The Aged) List Version 4”. Recommendations were not counted if a previously undocumented but clinically appropriate indication for the specific medication was identified until the discharge visit.

^b^ The recommendation to withdraw a medication rated as overuse was accepted. The medication was either directly stopped by the study physician or the recommendation was communicated to the patient's general practitioner.

^c^ Including nonsteroidal anti‐inflammatory drugs.

^d^ Including anxiolytics, hypnotics, antihistamines for systemic use (psycholeptics).

Table 6 shows the proportions of accepted recommendations from the advisory letter regarding underuse in the intervention group. Frequent indications for underuse were chronic pain, incontinence, and insomnia, with 66, 57, and 38 recommendations, respectively, whereas all other indications showed medication underuse in fewer than 15 study participants. Recommended initiation of treatment was accepted in more than half of the study participants for chronic pain and insomnia, whereas physicians only followed about one out of three recommendations for incontinence treatment.

**Table 6:** Proportions of accepted recommendations from the advisory letter regarding underuse in the intervention group per medication group or vaccine (*N* = 212; per‐protocol population).

	Recommendations^a^	Proportion accepted^b^
Indication for underuse	***n* (%)**	***n* (%)**
***Underuse of medications***		
Chronic pain	66	43 (65.2)
Incontinence	57	17 (29.8)
Insomnia	38	20 (52.6)
Hypothyroidism	14	7 (50.0)
Diabetes	10	4 (40.0)
Osteoporosis	9	8 (88.9)
COPD	8	4 (50.0)
Gastrointestinal illnesses	8	6 (75.0)
Stroke	5	1 (20.0)
Arterial hypertension	4	3 (75.0)
Constipation	4	2 (50.0)
Nausea and vomiting	2	1 (50.0)
Neutropenia	1	1 (100.0)
Atrial fibrillation	1	1 (100.0)
Anaemia, tumor‐related	1	1 (100.0)
Anaemia, Vitamin B12 or iron‐related	1	1 (100.0)
Bipolar disorder	1	1 (100.0)
Heart failure	1	0 (0.0)
**Total, medication‐related**	231	121 (52.4)
***Underuse of vaccinations***		
Covid‐19 vaccination	110	104 (94.5)
Herpes zoster vaccination	104	91 (87.5)
Pneumococcal vaccination	100	89 (89.0)
Influenza vaccination	65	53 (81.5)
**Total, vaccination‐related**	379	337 (89.9)
**Total, overall**	**610**	**458 (75.1)**

Abbreviation: COPD, chronic obstructive pulmonary disease.

^a^ Recommendations according to the “The FORTA (Fit fOR The Aged) List Version 4”. Recommendations were not counted if a previously undocumented medication for the specific indication was identified during the discharge visit.

^b^ The recommendation to initiate a new medication for an untreated indication was accepted by the study physician. The new medication was either directly initiated by the study physician or the recommendation was communicated to the patient's general practitioner.

All four recommended vaccinations for older adults were very commonly missing, with the annual Covid‐19 vaccination most frequently missing (in 110 out of 212 participants (52%)) and the annual influenza vaccination least commonly missing (in 65 out of 212 participants (31%)). The acceptance rate for all vaccination recommendations exceeded 80%.

## Discussion

### Summary of findings

This RCT demonstrated that an advisory letter containing the results of a comprehensive FORTA‐based medication review sent to the treating rehabilitation physician was highly feasible. The intervention statistically significantly reduced the total FORTA score (including vaccine underuse) among older cancer patients with polypharmacy from admission to discharge by approximately four score points compared to usual care. A reduction of four FORTA score points translates, for example, to the withdrawal of two PIMs, four drugs labeled overuse, or new treatments of four indications or taking four recommended vaccinations, which were previously missing. The acceptance rates among rehabilitation physicians of recommendations regarding PIM, overuse, medication, and vaccine underuse were comparably high and led to statistically significant reductions in the proportion of subjects exposed to all these highly common medication problems among older cancer patients during oncological rehabilitation.

### Comparison with previous studies

Our findings confirm the results from the two previous FORTA‐based RCTs in general older hospitalized patients, the VALFORTA trial [8] and its feasibility‐oriented pilot RCT [17]. They demonstrated the clinical applicability of the FORTA list, the acceptability of the recommendations across different indications, and a significant reduction in the FORTA score by (median) three points in the intervention group versus one point in the control group [8, 17]. Their median group difference of two points was slightly lower than the three points observed in our study (without vaccine underuse, which was not part of the first version of the FORTA list used in the VALFORTA trial). The difference can best be explained by the study populations. The older cancer patients in the intervention group of our study had a median FORTA score of four (without vaccine underuse), and the older hospitalized patients in the VALFORTA study's intervention group had a median FORTA score of three points. There was simply a higher need for improvements in the medication quality of geriatric cancer patients, and the OPTIMAL study showed that it was possible to achieve this goal with the pharmacist‐led counseling of rehabilitation physicians. In the VALFORTA study, the physicians conducted the medication reviews themselves.

Vaccine underuse was added to the FORTA list in 2021. Our trial was the first to evaluate the underuse of four recommended vaccinations in older cancer patients in Germany. The high acceptance rate (89.9%) for vaccine‐related recommendations observed in our intervention group was in line with that reported by a US‐American RCT with older cancer patients [18].

Furthermore, the statistically significant reductions in PIM and medication underuse align with previous evidence from RCTs across different clinical populations and medication assessment tools summarized in the systematic review by Cole et al. [19]. Importantly, unlike most successful RCTs included in this review, our intervention was not a typical medication review but rather a structured pharmaceutical care approach with algorithm‐assisted decision support and implementation left to the treating physician.

However, few RCTs with rather small sample sizes (*n* = 60–147) tested the feasibility and effectiveness of medication reviews in reducing medication problems in older cancer patients [18, 20, 21, 22], and their results are not directly comparable with others because the methods of the medication reviews differed.

### Acceptance of recommendations

The high acceptance rates of recommendations for PIM, overuse, and underuse of vaccinations (all >75%) by the rehabilitation physicians demonstrated the effectiveness of the FORTA list not only in detecting relevant medication problems but also in suggesting solutions that are mostly acceptable for the physicians. We consider the lower acceptance rate of 52% for underused drugs to be less clinically relevant. Among the top five FORTA indications, for which at least ten underuse‐specific recommendations were given, three displayed indications that can be treated non‐pharmacologically: Type 2 diabetes by lifestyle interventions, insomnia by sleep hygiene and behavioral therapy, and incontinence by bladder training [23, 24, 25].

### Implications for clinical practice

#### Clinical relevance of the total medication quality measured by the FORTA score

The OPTIMAL trial provides robust evidence for the effectiveness of FORTA‐based medication reviews in optimizing cancer and co‐medication quality for older cancer patients. The effect size is clinically relevant because the recorded reduction in the total FORTA score by approximately four score points (rounded) in the intervention compared to the control group translates to, for instance, the withdrawal of four drugs labeled overuse, the new initiation of four drugs for indications not yet treated, or the switch of two PIM to AMs. Evidence for the clinical relevance of treating medication overuse and underuse is rather weak [26, 27], but the evidence for the clinical relevance of reducing PIM is quite strong because PIM use has been linked to hospitalizations and premature death [2, 3, 7].

#### Clinical relevance of PIM use

Due to its frequency among older cancer patients and the associated risks, reducing PIM use appears to be particularly relevant. Thus, we advise physicians to critically review prescriptions for the most common PIM groups, including analgesics, beta blockers, antidepressants, antipsychotics, and sedatives, and to exploit appropriate alternative medications.

Nonsteroidal anti‐inflammatory drugs (NSAIDs) deserve particular attention as they accounted for almost two‐thirds (63%) of the pain‐related PIM. The recently published German guideline for pain management in geriatric patients also recommends the FORTA list to guide treatment selection and emphasizes the importance of carefully weighing the risks associated with NSAID use in older patients [28], which include cardiovascular events, renal toxicity, and gastrointestinal bleeding [29, 30, 31]. As this was often underacknowledged in clinical practice [32], we encourage physicians to give more consideration to recommended alternatives such as paracetamol, metamizole, and different opioids [5, 33].

To minimize inappropriate beta blocker use, we recommend that physicians first evaluate whether patients have any indication beyond arterial hypertension for their use, such as atrial fibrillation, heart failure, or previous acute coronary syndromes. For these indications, beta blockers would be classified as AMs [5, 33]. However, in isolated arterial hypertension, it is advisable to prioritize angiotensin‐converting‐enzyme inhibitors, angiotensin II receptor blockers, diuretics, and dihydropyridine calcium channel blockers, given the inferiority of beta blockers on clinical outcomes [34] and their more than seven‐fold increased odds for orthostatic hypotension [35] that may increase the risk for falls [36].

Anticholinergic antidepressants, antipsychotics, and sedatives are further common PIM groups that are associated with an elevated risk for falls [35, 36] as well as cognitive impairment [37]. Optimal treatment selection is essential to prevent these adverse drug events, and the FORTA list can help identify suitable alternatives depending on the underlying indication (e.g., depression, dementia‐related symptoms, and sleep disorders). Although PIM may be deemed necessary in individual cases, such as benzodiazepines in terminally ill cancer patients, the high acceptance rates for the recommended alternatives reflect that these PIMs are not necessary in most cases.

#### Clinical relevance of medication underuse

Analgesics, prolonged‐release melatonin against insomnia, and drugs against incontinence were among the most common underused medications. We identified most of these cases based on frequent and severe symptoms reported by patients in the self‐administered questionnaire. We assume that the physicians were often not aware of this symptom burden, as previous research demonstrated that pain and insomnia remain frequently undisclosed during medical consultations [38], and reporting incontinence can be a source of embarrassment. It is advisable for physicians to proactively ask about these symptoms because they have a large impact on the patient's quality of life. However, we acknowledge that it is worth exploring non‐pharmacological options first for insomnia and incontinence.

#### Clinical relevance of vaccination underuse

On average, two of the four vaccinations recommended for subjects 65 years or older in Germany were missing. Given that older cancer patients are an especially vulnerable patient group for infections, closing this gap is of high clinical relevance [39]. We recommend incorporating vaccination assessments into medication reviews. Furthermore, we encourage oncologists to proactively ask about vaccination status and not to leave this care only in the hands of the GP. The high acceptance rates of vaccine‐related recommendations by the physicians and their patients in our trial showed that it is possible to catch up on missing vaccinations in most cases.

#### Clinical relevance of medication overuse

PPIs and antithrombotic drugs were the most and third most common overused medication classes. Frequently, they are “leftovers” from hospital stays, where PPIs were prescribed to temporarily prevent medication‐induced upper gastrointestinal bleeding (e.g., through NSAIDs) and antithrombotic drugs to prevent major cardiovascular events for immobilized patients. They are usually not intended to be continued after hospital discharge [40, 41]. We recommend that physicians in rehabilitation clinics or the outpatient setting deprescribe these drugs in the absence of a clear indication [42]. Deprescribing PPIs is of special relevance for cancer patients as they may severely interact with antineoplastic agents, including proven loss of efficacy for certain tyrosine kinase inhibitors [43]. The second most frequent drug group with overuse was lipid‐lowering drugs, and in 79% of cases, physicians discontinued their use when notified about the overuse in the recommendation letter. The rehabilitation physicians had measurements of lipids and triglycerides from the admission blood samples at hand to base their decision on. It is advisable to always consider the potential discontinuation of lipid‐lowering drugs in clinical practice when these biomarkers are measured as part of routine check‐ups.

#### Application of the FORTA list in clinical practice

The FORTA list alone is not meant to replace clinical judgment and consideration of individual treatment goals. However, in combination with these standard medical procedures, no harm is expected from using it as a tool for guidance. The FORTA list is free of charge, easy to apply (via web or smartphone app) without specialized training, and available in many countries [44, 45]. These structural benefits would also support a potential large‐scale implementation into standard (rehabilitation) care in the future. The time required to conduct medication reviews manually depends on the number and complexity of morbidities and medications for the individual patient. Therefore, decentralized medication reviews, for example, with communication via advisory letters as conducted in our trial, represent a feasible strategy, especially when resources for medical and pharmaceutical personnel in the clinics are limited. Alternatively, an implementation through an automated PC‐based program could also be a viable, cost‐effective option. The software‐assisted medication reviews in our trial generally required < 15 min. Such software could enable clinicians to perform medication reviews without a pharmacist in most cases, as it incorporates the most relevant contraindications and drug–drug interactions.

### Strengths and limitations

To our knowledge, this was the first RCT to assess a medication review in the oncologic rehabilitation setting and the first to use the FORTA criteria in cancer patients. By combining physician‐ and patient‐reported information, we achieved a comprehensive overview of medical history (including untreated symptoms, such as pain, insomnia, incontinence, and constipation) and medication use (including over‐the‐counter medication use).

We are confident that our rigorous methodological approach reduced common biases (e.g., contamination bias by monitoring potential learning effects and sending sham recommendation letters for participants in the control group or selection bias by using an intention‐to‐treat approach as the main analysis). Given that our monitoring showed no change in how physicians treated patients in the control group over the course of the study, we can exclude that learning effects affected the results.

Although oncological rehabilitation may be organized differently in other countries, we are confident that results are generalizable to other developed countries, which have similar oncological care programs. The multi‐center study design with ten rehabilitation clinics located all over Germany and the subgroup analysis by clinics ensured that the results are robust and translatable into clinical practice in Germany.

The main limitation of this investigation is that clinicians could not be fully blinded because they could correctly guess the control group allocation of study participants for whom they received the sham advisory letter. However, we monitored the medication changes from rehabilitation clinic admission to discharge in the control group, and no relevant FORTA score improvements were detected.

## Conclusion

The pharmacist‐led comprehensive medication review with the FORTA score to counsel physicians in oncological rehabilitation wards was highly feasible and substantially improved the medication quality of older cancer patients with polypharmacy. Our findings support the use of structured, FORTA‐based medication reviews to identify and optimize PIM use, underuse, and overuse in this setting.

## Author contributions

*Conceptualization*: Ben Schöttker and Miriam Degen. *Methodology*: Ben Schöttker and Miriam Degen. *Resources*: Miriam Degen. *Investigation*: Miriam Degen and Ben Schöttker. *Data preparation*: Miriam Degen. *Formal analysis*: Miriam Degen. *Preparation of the manuscript*: Miriam Degen. *Review and editing*: all authors. *Supervision*: Ben Schöttker. All co‐authors accounted for reviewing and editing of the manuscript. Ben Schöttker and Miriam Degen are accountable for all aspects of the work in ensuring that questions related to the accuracy or integrity of any part of the work are appropriately investigated and resolved.

## Conflict of interest statement

V.G. reports support for the present manuscript through an enrollment fee per case received from the German Cancer Research Center; speaker honoraria from MSD, Lilly, and Novartis; being chair of the Working Group Geriatric Oncology DGHO/DGG/AIO and chair of the EHA Specialized Working Group on Aging and Hematology. B.L. reports being an alternate chair of the Verein für Rehabilitationsforschung Norderney (VFR). D.L. reports having received consulting fees, payment for expert testimony, and support for attending meetings and/or travel from Novartis, Onkowissen, Eli Lilly, Daiichi Sankyo, Gilead, and Stemline; payment or honoraria for lectures, presentations, speaker bureaus, manuscript writing, or educational events from Novartis, Pfizer, Eli Lilly, Daiichi Sankyo, Gilead, and Stemline. All other authors declare no conflict of interest. The funders had no role in study design, data collection and analysis, decision to publish, or preparation of the manuscript.

## Funding information

This study was supported by German Cancer Aid, Award No.: 70115079.

## Disclosure

The funder of the study had no role in study design, data collection, data analysis, data interpretation, or writing of the report.

## Supporting information




**Figure S1**. Distributions of total FORTA score at discharge and change in total FORTA score between admission and discharge, per‐protocol population (N = 418).
**Figure S2**. Distributions of FORTA sub‐scores at discharge and change in FORTA (sub‐)scores between admission and discharge, per‐protocol population (N = 418).
**Figure S3**. Flow chart of the study population.
**Figure S4**. Distribution of FORTA score and sub‐scores at admission and discharge, per‐protocol population (N = 418).
**Figure S5**. Distribution of change in FORTA score and sub‐scores from admission to discharge, per‐protocol population (N = 418).
**Table S1**. Proportion of missing data in intention‐to‐treat population (N = 443).
**Table S2**. FORTA (sub‐)scores and proportions exposed to medication problems at rehabilitation clinic admission in intention‐to‐treat (N = 443) and per‐protocol population (N = 418).
**Table S3**. Results of multivariate linear regression model with group allocation as independent and changes in mean difference in FORTA (sub‐)score scales between intervention and control group from admission to discharge as the dependent variable. Intention‐to‐treat population (N = 443).
**Table S4**. Multivariate linear regression model with cluster‐robust estimators for standard errors using the study physicians as the clustering variable, with group allocation as independent and changes in mean difference in FORTA (sub‐)score scales between intervention and control group from admission to discharge as the dependent variable. Intention‐to‐treat population (N = 443).
**Table S5**. Median difference in FORTA (sub‐)score scales between intervention and control group at discharge and mean difference in changes from admission to discharge in the per‐protocol population (N = 418).
**Table S6**. Proportions of study participants exposed to medication problems at discharge in the per‐protocol population (N = 418).
**Table S7**. Subgroup analysis by rehabilitation clinic for mean difference FORTA (sub‐)score change from admission to discharge, intention‐to‐treat population (N = 443).
**Table S8**. Subgroup analysis by sex for mean difference FORTA (sub‐)score change from admission to discharge, intention‐to‐treat population (N = 443).
**Table S9**. Subgroup analysis by age for mean difference FORTA (sub‐)score change from admission to discharge, intention‐to‐treat population (N = 443).

## Data Availability

The data will not be made available in an open access platform. After completion of the study, interested scientists can request data and can receive pseudonymized data upon approval of this application by the principal investigator of the OPTIMAL study (Prof. Dr. Ben Schöttker; b.schoettker@dkfz.de) and a signed data transfer agreement with the DKFZ.
