# Supplementary material for: Cancer co‐medication quality in older patients with polypharmacy: A large randomized trial testing a medication review versus usual care
Source: J Intern Med. 2026 Sep 23;300(6):802–20. doi: 10.1111/joim.70161 (PMC13741811; doi:10.1111/joim.70161)
Supplement: Supplementary file 1 [file JOIM-300-802-s001.docx]

**Supplementary Material to Article**

“Cancer co-medication quality in older patients with polypharmacy: a large randomized trial testing a medication review versus usual care”

Table of contents

[Figure S1. Distributions of total FORTA score at discharge and change in total FORTA score between admission and discharge, per-protocol population (N=418)	2](#_Toc234331928)

[Figure S2. Distributions of FORTA sub-scores at discharge and change in FORTA (sub-)scores between admission and discharge, per-protocol population (N=418)	3](#_Toc234331929)

[Table S1. Proportion of missing data in intention-to-treat population (N=443)	4](#_Toc234331930)

[Text S1. Details about the multiple imputation procedure	6](#_Toc234331931)

[Figure S3. Flow chart of the study population	8](#_Toc234331932)

[Table S2. FORTA (sub-)scores and proportions exposed to medication problems at rehabilitation clinic admission in intention-to-treat (N=443) and per-protocol population (N=418)	9](#_Toc234331933)

[Figure S4. Distribution of FORTA score and sub-scores at admission and discharge, per-protocol population (N=418)	10](#_Toc234331934)

[Figure S5. Distribution of change in FORTA score and sub-scores from admission to discharge, per-protocol population (N=418)	11](#_Toc234331935)

[Table S3. Results of multivariate linear regression model with group allocation as independent and changes in mean difference in FORTA (sub-)score scales between intervention and control group from admission to discharge as the dependent variable. Intention-to-treat population (N=443)	12](#_Toc234331936)

[Table S4. Multivariate linear regression model with cluster-robust estimators for standard errors using the study physicians as the clustering variable, with group allocation as independent and changes in mean difference in FORTA (sub-)score scales between intervention and control group from admission to discharge as the dependent variable. Intention-to-treat population (N=443)	13](#_Toc234331937)

[Table S5. Median difference in FORTA (sub-)score scales between intervention and control group at discharge and mean difference in changes from admission to discharge in the per-protocol population (N=418)	14](#_Toc234331938)

[Table S6. Proportions of study participants exposed to medication problems at discharge in the per-protocol population (N=418)	15](#_Toc234331939)

[Table S7. Subgroup analysis by rehabilitation clinic for mean difference FORTA (sub-)score change from admission to discharge, intention-to-treat population (N=443)	16](#_Toc234331940)

[Table S8. Subgroup analysis by sex for mean difference FORTA (sub-)score change from admission to discharge, intention-to-treat population (N=443)	17](#_Toc234331941)

[Table S9. Subgroup analysis by age for mean difference FORTA (sub-)score change from admission to discharge, intention-to-treat population (N=443)	17](#_Toc234331942)

[References	18](#_Toc234331943)

# Figure S1. Distributions of total FORTA score at discharge and change in total FORTA score between admission and discharge, per-protocol population (N=418)

Abbreviations: FORTA, Fit fOR The Aged List Version 4.

# Figure S2. Distributions of FORTA sub-scores at discharge and change in FORTA (sub-)scores between admission and discharge, per-protocol population (N=418)

Abbreviations: PIM, potentially inappropriate medication; FORTA, Fit fOR The Aged List Version 4.

# Table S1. Proportion of missing data in intention-to-treat population (N=443)

| **Variable** | **N (data)** | **Proportion missing (%)** |
| --- | --- | --- |
| *Sociodemographic/ -economic factors* |  |  |
| Sex | 443 | 0.0 |
| Age | 443 | 0.0 |
| Years of schooling | 440 | 0.7 |
| Partnership | 443 | 0.0 |
| Social support^a^ | 438 | 1.1 |
| *Lifestyle factors* |  |  |
| BMI *(kg/m^2^)* | 440 | 0.7 |
| Alcohol consumption *(g/day)* | 432 | 2.5 |
| Physical activity^b^ | 440 | 0.7 |
| Smoking status | 440 | 0.7 |
| *Cancer* |  |  |
| Cancer site | 443 | 0.0 |
| Stage | 388 | 12.4 |
| Cancer therapy |  |  |
| Chemotherapy | 435 | 1.8 |
| Radiotherapy | 436 | 1.6 |
| Surgery | 436 | 1.6 |
| *Comorbidities* |  |  |
| Diabetes | 443 | 0.0 |
| Arterial hypertension | 443 | 0.0 |
| Stroke | 443 | 0.0 |
| Myocardial infarction | 443 | 0.0 |
| Heart failure | 443 | 0.0 |
| Acute coronary syndromes | 443 | 0.0 |
| Atrial fibrillation | 443 | 0.0 |
| Asthma, COPD | 443 | 0.0 |
| Osteoporosis | 443 | 0.0 |
| Chronic pain | 443 | 0.0 |
| Depression | 443 | 0.0 |
| Insomnia | 443 | 0.0 |
| Gastrointestinal illnesses | 443 | 0.0 |
| Incontinence | 443 | 0.0 |
| Anaemia, iron or B12-related | 443 | 0.0 |
| Constipation | 443 | 0.0 |
| Hypothyroidism | 443 | 0.0 |
| Chronic kidney disease | 443 | 0.0 |
| Dyslipidaemia | 443 | 0.0 |
| Gout | 443 | 0.0 |
| Peripheral vascular disease | 443 | 0.0 |
| *Physical/diagnostic assessments* |  |  |
| Depressive symptoms^c^ | 440 | 0.7 |
| Frailty^d^ | 432 | 2.5^e^ |
| Global quality of life score^f^ | 441 | 0.2 |

Abbreviations: BMI, body mass index; COPD, chronic obstructive pulmonary disease.

^a^ According to Duke-UNC Functional Social Support Questionnaire 5-item version.
^b^ According to Rapid Assessment of Physical Activity (RAPA) tool.
^c^ According Geriatric Depression Scale.
^d^ According to FRAIL scale.
^e^ Comprised 5 domains which were imputed separately. Maximum proportion of missing values: 2.5%. Minimum proportion of missing values: 0.0%.
^f^ According to the EORTC QLQ-C30 global health status /quality of life scale.

# Text S1. Details about the multiple imputation procedure

Prior to imputation, we assessed missingness patterns visually using a grid display, which revealed an arbitrary pattern, and explored potential sources of missingness. Since the distribution of endpoint values appeared to depend on the study arm, the imputation model was stratified by study arm.

To develop the imputation model, a predefined set of 50 candidate variables was established, which, in accordance with recommended practice [1, 2], included variables (potentially) related to the missingness and variance (based on explorative assessments of missing patterns) or to the outcomes (based on clinical relevance and existing literature), as well as the outcomes. In detail, the following variables were included: age, sex, study centre, years of schooling, partnership, social support, body mass index, daily alcohol consumption, physical activity, smoking status, cancer site, cancer stage, cancer surgery, chemotherapy, radiotherapy, 22 comorbidities as single variables (including diabetes, arterial hypertension, stroke, myocardial infarction, heart failure, acute coronary syndromes, atrial fibrillation, asthma/COPD, osteoporosis, chronic pain, depression, insomnia, gastrointestinal illnesses, incontinence, anaemia, constipation, hypothyroidism, chronic kidney disease, dyslipidaemia, peripheral vascular disease, dementia, and neutropenia), 5 frailty sub-scales as single variables (fatigue, resistance, ambulation, illnesses, and loss of weight), global quality of life, depressive symptoms, FORTA sub-scores (PIM use, overuse, underuse without vaccines, number of received vaccines) at admission and discharge.

Multicollinearity and model stability were examined before imputation using the variance inflation factor (VIF). After exclusion of two comorbidities (neutropenia and dementia), all variables had VIF < 8 and were included in the final imputation model without interaction terms.

We performed the SAS procedure ‘PROC MI’ with the Markov Chain Monte Carlo (MCMC) method with 200 burn-in iterations and 1000 iterations stratified by study arm.

The performance and goodness of fit of the imputation model were thoroughly assessed through trace plots, autocorrelation plots, and comparisons of distributions before and after imputation, in line with recommended practice [1, 3], indicating good convergence, adequate model performance, and plausible imputations.

To calculate analytical outcomes, we aggregated the imputed datasets with the SAS procedure ‘PROC MIANALYZE’.

# Figure S3. Flow chart of the study population

# Table S2. FORTA (sub-)scores and proportions exposed to medication problems at rehabilitation clinic admission in intention-to-treat (N=443) and per-protocol population (N=418)

|  | **Intention-to-treat population (N=443)** | | |  | **Per-protocol population (N=418)** | | |
| --- | --- | --- | --- | --- | --- | --- | --- |
| **Exposure** | **Score** |  | **Exposed** |  | **Score** |  | **Exposed** |
|  | **Median (IQR)** |  | **N (%)^a^** |  | **Median (IQR)** |  | **N (%)^a^** |
| **Total FORTA score^a^** |  |  |  |  |  |  |  |
| Incl. vaccine underuse | 6 (4; 8) |  | 443 (100.0) |  | 6 (4; 8) |  | 418 (100.0) |
| Without vaccine underuse | 4 (3; 6) |  | 433 (97.7) |  | 4 (3; 6) |  | 410 (98.1) |
| **FORTA score sub-categories** |  |  |  |  |  |  |  |
| PIM | 2 (0; 4) |  | 293 (66.1) |  | 2 (0; 4) |  | 277 (66.3) |
| Overuse | 1 (0; 1) |  | 261 (58.9) |  | 1 (0; 1) |  | 248 (59.3) |
| Underuse |  |  |  |  |  |  |  |
| Incl. vaccine underuse | 3 (2; 4) |  | 419 (94.6) |  | 3 (2; 4) |  | 394 (94.3) |
| Without vaccine underuse | 1 (0; 2) |  | 315 (71.1) |  | 1 (0; 2) |  | 298 (71.3) |

Abbreviations: CI, confidence interval; FORTA, FORTA, Fit fOR The Aged List Version 4; IQR, interquartile range; PIM, potentially inappropriate medication.

^a^ Recommendations according to the “The FORTA (Fit fOR The Aged) List Version 4”.

# Figure S4. Distribution of FORTA score and sub-scores at admission and discharge, per-protocol population (N=418)

Abbreviations: PIM, potentially inappropriate medication; FORTA, Fit fOR The Aged List Version 4.

# Figure S5. Distribution of change in FORTA score and sub-scores from admission to discharge, per-protocol population (N=418)

Abbreviations: PIM, potentially inappropriate medication; FORTA, Fit fOR The Aged List Version 4.

# Table S3. Results of multivariate linear regression model with group allocation as independent and changes in mean difference in FORTA (sub-)score scales between intervention and control group from admission to discharge as the dependent variable. Intention-to-treat population (N=443)

| **FORTA score change^a^** | **ß coefficient (95% CI)^b^** | **p** |
| --- | --- | --- |
| **Total** |  |  |
| Incl. vaccines | **-3.8 (-4.2; -3.3)** | **<.001** |
| Without vaccines | **-2.6 (-3.0; -2.3)** | **<.001** |
| **Sub-scores** |  |  |
| PIM | **-1.5 (-1.8; -1.2)** | **<.001** |
| Overuse | **-0.6 (-0.7; -0.5)** | **<.001** |
| Underuse |  |  |
| Underuse incl. vaccines | **-1.7 (-1.9; -1.4)** | **<.001** |
| Underuse without vaccines | **-0.5 (-0.7; -0.4)** | **<.001** |

Values in bold are statistically significant (p<0.05).
Abbreviations: CI, confidence interval; FORTA, Fit fOR The Aged List Version 4; PIM, potentially inappropriate medication.

^a^ Recommendations according to the “The FORTA (Fit fOR The Aged) List Version 4”.
^b^ Model adjusted for baseline FORTA score or the respective FORTA sub-score, age, sex, education, partnership, social support, body mass index, alcohol consumption, physical activity, smoking status, cancer site, cancer stage, previous cancer therapy, comorbidities as single covariates (including diabetes, arterial hypertension, stroke, myocardial infarction, heart failure, acute coronary syndromes, atrial fibrillation, asthma/COPD, osteoporosis, chronic pain, depression, insomnia, gastrointestinal illnesses, incontinence, anaemia, constipation, hypothyroidism, chronic kidney disease, dyslipidaemia, peripheral vascular disease), frailty, global quality of life, and depressive symptoms.

# Table S4. Multivariate linear regression model with cluster-robust estimators for standard errors using the study physicians as the clustering variable, with group allocation as independent and changes in mean difference in FORTA (sub-)score scales between intervention and control group from admission to discharge as the dependent variable. Intention-to-treat population (N=443)

| **FORTA score change^a^** | **ß coefficient (95% CI)^b,c^** | **p** |
| --- | --- | --- |
| **Total** |  |  |
| Incl. vaccines | **-3.7 (-5.0; -2.4)** | **<.001** |
| Without vaccines | **-2.6 (-3.6; -1.6)** | **<.001** |
| **Sub-scores** |  |  |
| PIM | **-1.5 (-2.0; -0.9)** | **<.001** |
| Overuse | **-0.6 (-0.8; -0.4)** | **<.001** |
| Underuse |  |  |
| Underuse incl. vaccines | **-1.6 (-2.3; -1.0)** | **<.001** |
| Underuse without vaccines | **-0.5 (-0.8; -0.3)** | **<.001** |

Values in bold are statistically significant (p<0.05).
Abbreviations: CI, confidence interval; FORTA, Fit fOR The Aged List Version 4; PIM, potentially inappropriate medication.

^a^ Recommendations according to the “The FORTA (Fit fOR The Aged) List Version 4”.
^b^ Model adjusted for baseline FORTA score or the respective FORTA sub-score, age, sex, education, partnership, social support, body mass index, alcohol consumption, physical activity, smoking status, cancer site, cancer stage, previous cancer therapy, comorbidities as single covariates (including diabetes, arterial hypertension, stroke, myocardial infarction, heart failure, acute coronary syndromes, atrial fibrillation, asthma/COPD, osteoporosis, chronic pain, depression, insomnia, gastrointestinal illnesses, incontinence, anaemia, constipation, hypothyroidism, chronic kidney disease, dyslipidaemia, peripheral vascular disease), frailty, global quality of life, and depressive symptoms.
^c^ Cluster-robust standard errors were estimated using the study physicians as the clustering variable. Study physicians who recruited < 1 participant in either of the two study arms were summarized into one cluster. A total of 23 different physicians recruited participants (n_control;_ n_intervention_): physician 1 (1; 2); physician 2 (2; 4); physician 3 (7; 6); physician 4 (63; 65); physician 5 (4; 5); physician 6 (1; 3); physician 7 (66; 72); physician 8 (1; 0); physician 9 (0; 1); physician 10 (0; 12); physician 11 (0; 1); physician 12 (0; 1); physician 13 (4; 3); physician 14 (1; 0); physician 15 (0; 1); physician 16 (40; 41); physician 17 (5; 4); physician 18 (0; 1); physician 19 (0; 1) physician 20 (6; 5); physician 21 (1; 1); physician 22 (3; 0); physician 23 (4; 5). Thus, physicians 1, 2, 3, 4, 5, 6, 7, 13, 16, 17, 20, 21, and 23 were used as 13 single clusters, and all other physicians were allocated to the 14^th^ cluster.

# Table S5. Median difference in FORTA (sub-)score scales between intervention and control group at discharge and mean difference in changes from admission to discharge in the per-protocol population (N=418)

| **FORTA score^a^** |  | **At Discharge** | | | |  | **Change from Admission to Discharge** | | | |
| --- | --- | --- | --- | --- | --- | --- | --- | --- | --- | --- |
|  |  | **Control (n=206) Median (IQR)** | **Intervention (n=212) Median (IQR)** | **Median  Difference (95% CI)** | **p** |  | **Control (n=206) Mean (95% CI)** | **Intervention (n=212) Mean (95% CI)** | **Difference  Mean (95% CI)** | **p** |
| **Total** |  |  |  |  |  |  |  |  |  |  |
| Incl. vaccines |  | 6 (4; 9) | 1 (0; 3) | **-5 (-5.7; -4.3)** | **<.001** |  | -0.1 (-0.3; 0.1) | **-4.0 (-4.4; -3.5)** | **-3.8 (-4.3; -3.4)** | **<.001** |
| Without vaccines |  | 4 (3; 6) | 1 (0; 3) | **-3 (-3.3; -2.7)** | **<.001** |  | 0.2 (0.1; 0.4) | **-2.4 (-2.8; -2.1)** | **-2.7 (-3.0; -2.3)** | **<.001** |
| **Sub-scores** |  |  |  |  |  |  |  |  |  |  |
| PIM |  | 2 (0; 4) | 0 (0; 2) | **-2 (-2.0; -2.0)** | **<.001** |  | 0.0 (-0.1; 0.1) | **-1.5 (-1.8; -1.1)** | **-1.5 (-1.8; -1.1)** | **<.001** |
| Overuse |  | 1 (0; 2) | 0 (0; 1) | **-1 (-1.0; -1.0)** | **<.001** |  | 0.1 (0.1; 0.2) | **-0.5 (-0.6; -0.4)** | **-0.6 (-0.8; -0.5)** | **<.001** |
| Underuse |  |  |  |  |  |  |  |  |  |  |
| Incl. vaccine underuse |  | 3 (1; 4) | 1 (0; 1) | **-2 (-2.8; -1.2)** | **<.001** |  | -0.3 (-0.4; -0.1) | **-2.0 (-2.2; -1.6)** | **-1.7 (-2.0; -1.4)** | **<.001** |
| Without vaccine underuse |  | 1 (0; 2) | 0 (0; 1) | **-1 (-1.0; -1.0)** | **<.001** |  | 0.1 (0.0; 0.2) | **-0.4 (-0.6; -0.3)** | **-0.6 (-0.7; -0.4)** | **<.001** |

Values in bold are statistically significant (p<0.05).
Abbreviations: CI, confidence interval; FORTA, Fit fOR The Aged List Version 4; IQR, interquartile range; PIM, potentially inappropriate medication.

^a^ Recommendations according to the “The FORTA (Fit fOR The Aged) List Version 4”.

# Table S6. Proportions of study participants exposed to medication problems at discharge in the per-protocol population (N=418)

| **Medication problems^a^** |  | **Control (n=206) N (%)** | **Intervention (n=212) N (%)** | **Absolute difference**^b^ **(95% CI)** | **p** |
| --- | --- | --- | --- | --- | --- |
| **Any (FORTA score > 0)** |  |  |  |  |  |
| Incl. vaccines |  | 206 (100.0) | 155 (73.1) | **-26.9 (-32.9; -20.9)** | **<.001**^c^ |
| Without vaccines |  | 206 (100.0) | 152 (71.7) | **-28.3 (-34.4; -22.2)** | **<.001**^c^ |
| **FORTA sub-categories** |  |  |  |  |  |
| PIM |  | 145 (70.4) | 56 (26.4) | **-44.0 (-52.6; -35.4)** | **<.001** |
| Overuse |  | 133 (64.6) | 68 (32.1) | **-32.5 (-41.6; -23.4)** | **<.001** |
| Underuse |  |  |  |  |  |
| Incl. vaccines |  | 184 (89.3) | 110 (51.9) | **-37.4 (-45.4; -29.5)** | **<.001** |
| Without vaccines |  | 150 (72.8) | 95 (44.8) | **-28.0 (-37.0; -19.0)** | **<.001** |

Values in bold are statistically significant (p<0.05).
Abbreviations: CI, confidence interval; FORTA, Fit fOR The Aged List Version 4, PIM, potentially inappropriate medication.

^a^ Recommendations according to the “The FORTA (Fit fOR The Aged) List Version 4”.
^b^ In proportions exposed to the relevant medication problem.
^c^ Tested with Fisher’s exact test, as all participants of the control group were exposed.

# Table S7. Subgroup analysis by rehabilitation clinic for mean difference FORTA (sub-)score change from admission to discharge, intention-to-treat population (N=443)

| **Subgroups** |  |  | **Control** | |  | **Intervention** | |  | **Difference** | **p** |
| --- | --- | --- | --- | --- | --- | --- | --- | --- | --- | --- |
|  |  |  | **n** | **Mean (95% CI)** |  | **n** | **Mean (95% CI)** |  | **Mean (95% CI)** |  |
| ***Clinic in Ückeritz*** |  |  | 68 | -0.4 (-0.7; 0.0) |  | 76 | -5.3 (-6.0; -4.6) |  | **-4.9 (-5.7; -4.1)** | **<.001** |
| ***Clinic in Durbach*** |  |  | 65 | 0.2 (-0.1; 0.6) |  | 68 | -4.5 (-5.1; -4.0) |  | **-4.7 (-5.4; -4.1)** | **<.001** |
| ***Clinic in Rüdersdorf*** |  |  | 41 | -0.5 (-0.9; -0.1) |  | 40 | -2.4 (-3.2; -1.6) |  | **-1.9 (-2.8 -1.0)** | **<.001** |
| ***Other clinics^a^*** |  |  | 36 | 0.0 (-0.6; 0.6) |  | 49 | -2.0 (-2.9; -1.1) |  | **-2.0 (-3.1; -0.9)** | **<.001** |

^a^ Clinics with <50 participants were summarized. Clinic (n_control;_ n_intervention_): clinic in Bad Neuenahr-Ahrweiler (17; 18); clinic in Blankenburg (0; 0); clinic in Cologne (4; 3); clinic in Buckow (1; 3); clinic in Bad Salzuflen (13; 12); clinic in Bad Wildungen (0; 1); and clinic in Bad Lippenspringe (1; 12).

Note: A total of 23 different physicians recruited participants (n_control;_ n_intervention_): physician 1 (1; 2); physician 2 (2; 4); physician 3 (7; 6); physician 4 (63; 65); physician 5 (4; 5); physician 6 (1; 3); physician 7 (66; 72); physician 8 (1; 0); physician 9 (0; 1); physician 10 (0; 12); physician 11 (0; 1); physician 12 (0; 1); physician 13 (4; 3); physician 14 (1; 0); physician 15 (0; 1); physician 16 (40; 41); physician 17 (5; 4); physician 18 (0; 1); physician 19 (0; 1) physician 20 (6; 5); physician 21 (1; 1); physician 22 (3; 0); physician 23 (4; 5).

# Table S8. Subgroup analysis by sex for mean difference FORTA (sub-)score change from admission to discharge, intention-to-treat population (N=443)

| **Subgroups** |  |  | **Control** | |  | **Intervention** | |  | **Difference** | **p** |
| --- | --- | --- | --- | --- | --- | --- | --- | --- | --- | --- |
|  |  |  | **n** | **Mean (95% CI)** |  | **n** | **Mean (95% CI)** |  | **Mean (95% CI)** |  |
| ***Females*** |  |  | 147 | -0.2 (-0.4; 0.0) |  | 155 | -4.0 (-4.5; -3.5) |  | **-3.8 (-4.4; -3.2)** | **<.001** |
| ***Males*** |  |  | 62 | 0.0 (-0.4; 0.3) |  | 79 | -3.6 (-4.2; -3.0) |  | **-3.6 (-4.3; -2.8)** | **<.001** |

Values in bold are statistically significant (p<0.05).
Abbreviations: CI, confidence interval.

# Table S9. Subgroup analysis by age for mean difference FORTA (sub-)score change from admission to discharge, intention-to-treat population (N=443)

| **Subgroups** |  |  | **Control** | |  | **Intervention** | |  | **Difference** | **p** |
| --- | --- | --- | --- | --- | --- | --- | --- | --- | --- | --- |
|  |  |  | **n** | **Mean (95% CI)** |  | **n** | **Mean (95% CI)** |  | **Mean (95% CI)** |  |
| ***65 – 74 years*** |  |  | 124 | -0.2 (-0.5; 0.1) |  | 147 | -4.1 (-4.7; 3.6) |  | **-3.9 (-4.5; -3.3)** | **<.001** |
| ***75 – 92 years*** |  |  | 85 | 0.0 (-0.3; 0.2) |  | 87 | -3.4 (-4.0; -2.8) |  | **-3.4 (-4.1; -2.7)** | **<.001** |

Values in bold are statistically significant (p<0.05).
Abbreviations: CI, confidence interval.

# References

1.	van Buuren S. *Flexible Imputation of Missing Data*, 2nd edn. New York: Chapman & Hall/CRC; 2018.

2.	Moons KG, Donders RA, Stijnen T, Harrell FE, Jr. Using the outcome for imputation of missing predictor values was preferred. *J Clin Epidemiol* 2006, **59**(10):1092-1101. doi: 10.1016/j.jclinepi.2006.01.009

3.	Abayomi K, Gelman A, Levy M. Diagnostics for Multivariate Imputations. *Journal of the Royal Statistical Society Series C: Applied Statistics* 2008, **57**(3):273-291. doi: 10.1111/j.1467-9876.2007.00613.x
